# Dbx1b defines the dorsal habenular progenitor domain in the zebrafish epithalamus

**DOI:** 10.1186/1749-8104-9-20

**Published:** 2014-09-12

**Authors:** Benjamin J Dean, Begum Erdogan, Joshua T Gamse, Shu-Yu Wu

**Affiliations:** 1 Department of Biological Sciences, Vanderbilt University, Box 351634 Station B, Nashville, TN 37235-1634, USA; 2 Program in Neuroscience, Vanderbilt University, Nashville, TN 37232, USA

## Abstract

**Background:**

The conserved habenular nuclei function as a relay system connecting the forebrain with the brain stem. They play crucial roles in various cognitive behaviors by modulating cholinergic, dopaminergic and serotonergic activities. Despite the renewed interest in this conserved forebrain region because of its importance in regulating aversion and reward behaviors, the formation of the habenular nuclei during embryogenesis is poorly understood due to their small size and deep location in the brain, as well as the lack of known markers for habenular progenitors. In zebrafish, the bilateral habenular nuclei are subdivided into dorsal and ventral compartments, are particularly large and found on the dorsal surface of the brain, which facilitates the study of their development.

**Results:**

Here we examine the expression of a homeodomain transcription factor, *dbx1b,* and its potential to serve as an early molecular marker of dorsal habenular progenitors. Detailed spatiotemporal expression profiles demonstrate that the expression domain of *dbx1b* correlates with the presumptive habenular region, and *dbx1b*-expressing cells are proliferative along the ventricle. A lineage-tracing experiment using the Cre-lox system confirms that all or almost all dorsal habenular neurons are derived from *dbx1*b-expressing cells. In addition, mutant analysis and pharmacological treatments demonstrate that both initiation and maintenance of *dbx1b* expression requires precise regulation by fibroblast growth factor (FGF) signaling.

**Conclusions:**

We provide clear evidence in support of *dbx1b* marking the progenitor populations that give rise to the dorsal habenulae. In addition, the expression of *dbx1b* in the dorsal diencephalon is tightly controlled by FGF signaling.

## Background

The habenular nuclei (habenulae) develop in the dorsal diencephalon of vertebrates. These bilaterally paired nuclei receive inputs from the limbic system and basal ganglia and send outputs to dopaminergic and serotonergic centers. Despite their small size, these nuclei play crucial roles in regulating aversion and reward behaviors [1]. Moreover, the fact that the habenulae are a nexus for monoamine circuits highlights the importance of this brain region for studies of neuromodulation and multi-circuit integration.

The habenular nuclei can be divided into two functionally distinct subnuclei alternately referred to as medial and lateral (mammals) or dorsal and ventral (zebrafish) subnuclei. Recent study of the habenulae has largely focused on the ventral habenulae, but interest in the dorsal habenulae is increasing [2]. Alterations in dorsal habenular structure and function during development have been linked to dopamine receptor expression as well as impulsivity, attention, aversion and spatial memory endophenotypes in rodents [3,4]. In zebrafish, the dorsal habenulae regulate fear and aversion [5,6]. Together these studies implicate the dorsal habenulae as a possible mediator of attention deficit hyperactivity disorder (ADHD), depression and anxiety [2]. Interestingly, the dorsal habenulae are also sites of intense acetylcholine receptor and transporter expression and control nicotine intake [7-9]. This raises the possibility of targeting the habenulae as a therapeutic intervention to nicotine addiction. Beyond translational research, the zebrafish habenulae also serve as an excellent model to study the basic mechanisms underlying the development of left-right brain asymmetry. Unlike the mammalian habenulae, teleost dorsal habenulae are robustly asymmetric in anatomy, gene expression and functional connectivity [10].

Interest in how the dorsal habenulae integrate into cholinergic and monoaminergic circuitry has put pressure on researchers to understand dorsal habenular development. Indeed, Beretta *et al*. [11] have shown that dorsal and ventral habenular neurons arise from distinct progenitor populations. While progress has been made in describing habenular neurogenesis, differentiation and elaboration of processes, there are no known markers for habenular progenitors [12-16]. Therefore, finding marker genes that label dorsal habenular progenitors will be fundamental to studying how the diverse set of dorsal habenular neurons are generated and integrated into neural circuits underpinning aversive behavior as well as pathological addictive and depressive behaviors.

The *dbx* homeodomain transcription factors play a central role in regulating progenitor status in several regions in the developing central nervous system, including the spinal cord [17,18]. However, the upstream regulatory pathways that regulate *dbx* gene-family expression are not known. Here we report that in zebrafish, *dbx1b* is expressed in the dorsal diencephalon where it marks dorsal habenular progenitors, and further, that dorsal diencephalic expression of *dbx1b* is controlled by fibroblast growth factor (FGF) signaling.

## Results and discussion

### *Dbx1b* is expressed in the presumptive habenulae

During our ongoing efforts to characterize transcription factors (TFs) that are expressed in the dorsal diencephalic region between 24 and 48 hours post-fertilization (hpf), we focused on a family of homeodomain-containing TFs encoded by the *dbx* genes because of their known roles in neural progenitors. There are three *dbx* genes in the zebrafish genome, and we carefully examined the expression pattern of the two *dbx1* paralogs, *dbx1a* and *dbx1b*. We excluded *dbx2* from our study because its expression has been detailed previously [19]. At 28 hpf, *dbx1a* and *dbx1b* showed similar yet distinct expression patterns (Additional file 1). As shown previously [20], *dbx1a* was expressed in sharply restricted domains in the diencephalon with prominent expression in the prethalamus and thalamus (Additional file 1: panel A). Expression of *dbx1a* and *dbx1b* was similar in the prethalamic region, but in the thalamic region *dbx1b* was expressed at a much lower level than *dbx1a* (Additional file 1; panel C). A more striking difference between the patterns of these two paralogs was the expression of *dbx1b* in the dorsal diencephalon, where *dbx1a* was completely absent (compare arrowheads in Additional file 1). Expression of *dbx1b* was excluded from the *otx5*-positive pineal complex, the other major structure of the dorsal diencephalon (Additional file 1, panel C). These data suggested that *dbx1b* could be an early molecular marker for the presumptive habenulae.

**Figure 1:**
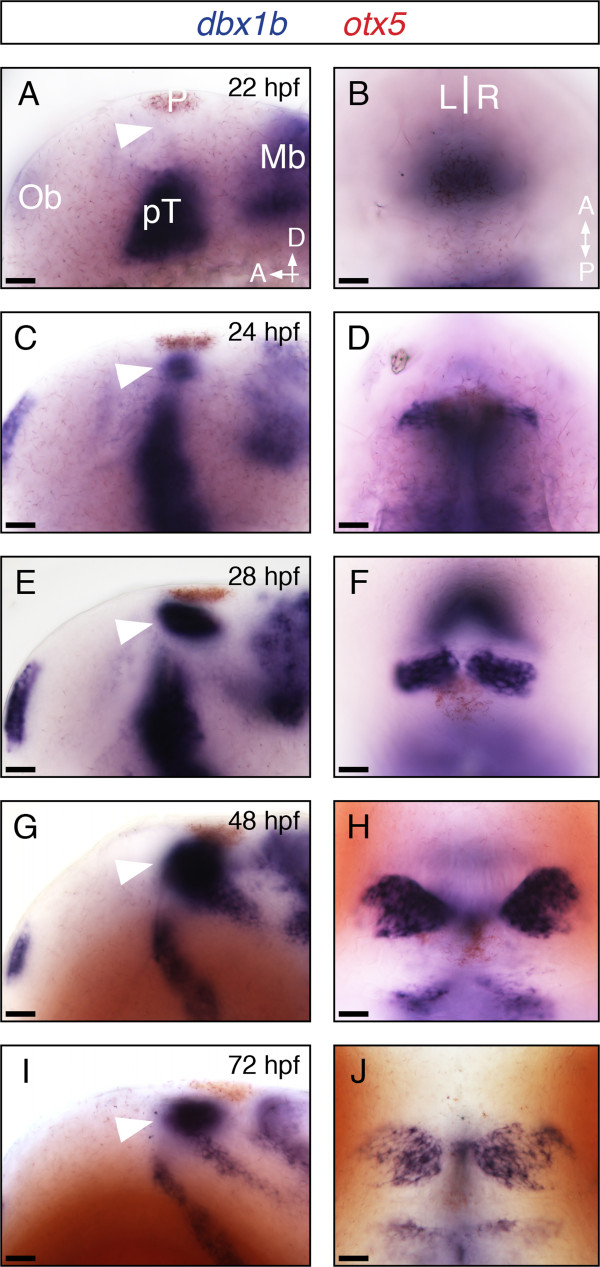
***Dbx1b *is expressed throughout habenular development. (A-J)** Lateral and dorsal views of *dbx1b* expression (blue) during early brain development. Dorsal diencephalic expression of *dbx1b* appeared shortly after 22 hpf and continued through 72 hpf (arrowhead). *otx5* (red) marks the pineal complex (P). Ob, olfactory bulb; pT, prethalamus; Mb, midbrain. Scale bars are 10 μm.

Next, we closely examined the expression of *dbx1b* at different developmental stages (Figure 1). At 22 hpf, expression of *dbx1b* was not yet present in the presumptive habenular region, although the prethalamic and midbrain regions showed strong expression (Figure 1A,B). By 24 hpf, habenular *dbx1b* expression appeared (Figure 1C,D) and was maintained through at least 96 hpf (Figure 1E-J and data not shown). Moreover, *dbx1b* expression remained highest adjacent to the third ventricle of the brain, and was absent from regions distal to the ventricle by 48 hpf (Figure 1H and 1J; dorsal views). Because neuronal progenitors are often found in regions adjacent to the ventricle in the developing brain, these data led us to speculate that the *dbx1b* expression pattern includes the progenitors of habenular neurons.

### *Dbx1b* labels dorsal habenular progenitors

**Figure 2:**
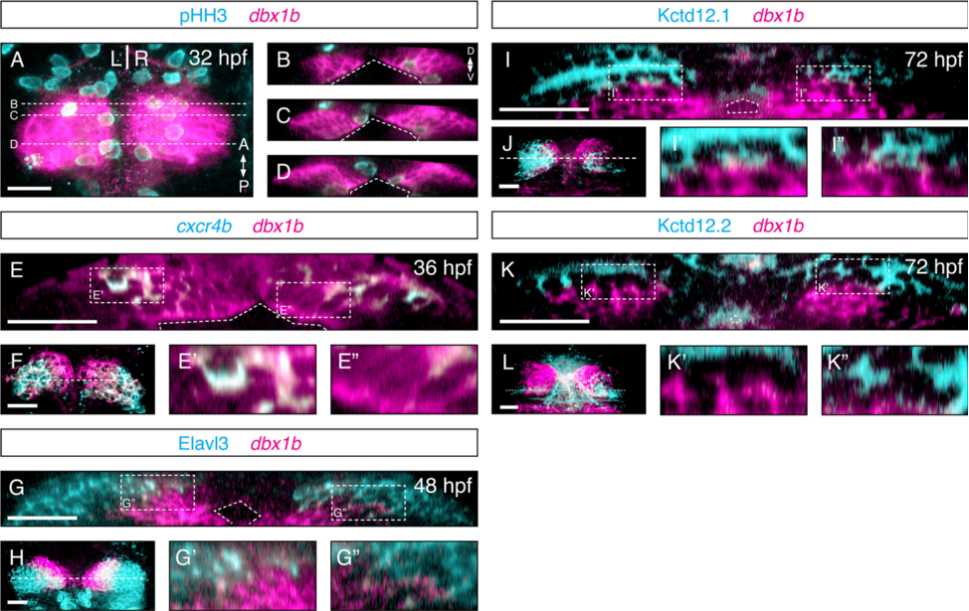
***Dbx1b *marks a proliferative periventricular domain in the epithalamus. (A)** A dorsal view of the epithalamus showed *dbx1b* and phosphohistone H3 (pHH3) expression. **(B-D)** Coronal optical sections revealed that *dbx1b*-positive cells are pHH3-positive. **(E)** A presumptive habenular precursor marker, *cxcr4b*, showed partial overlaps with *dbx1b*. Significantly, the co-expression domain (E’) was more dorsolateral while the *dbx1b*-only domain (E”) was along the ventricle. **(F)** A dorsal view of *dbx1b* and *cxcr4b* co-expression. **(G-G”)***dbx1b* expression showed very little overlap with the neuronal marker Elav3l. **(H)** A dorsal view of *dbx1b-* and Elav3l-expressing domains. **(I-I” and K-K”)** No overlapped expression was observed between *dbx1b* and markers of differentiated habenular neurons, Kctd12.1 and Kctd12.2. **(J and L)** Dorsal views of *dbx1b-* and Kctd12.1/12.2-expressing domains. The ventricle is marked by angled dashed lines. Insets are shown with dashed rectangles. Scale bars are 50 μm.

To support the hypothesis that *dbx1b*-expressing cells represent habenular progenitors, we examined if these cells are proliferative. Indeed, as shown by phospho-histone H3 staining at 32 hpf, the *dbx1b*-positive cells close to the ventricular surface were proliferative, which was consistent with progenitor cell identity (Figure 2A-D). To see if *dbx1b* expression is restricted to progenitors we compared the expression of previously described precursor and neuronal habenular markers. *Cxcr4b* has been proposed as a marker of progenitors as well as post-mitotic habenular precursors [12]. At 36 hpf, a subset of *dbx1b*-expressing cells co-expressed *cxcr4b*. Specifically, *cxcr4b* expression was restricted to the dorsal half of the *dbx1b* expression domain leaving a ventral region of *dbx1b*-only expression along the ventricle (Figure 2E-F). At 48 hpf, Elavl3 (also known as HuC) marks post-mitotic neurons. Expression of Elavl3 and *dbx1b* was more distinct than *cxcr4b* and *dbx1b*, with Elavl3 expressed dorsally and laterally while *dbx1b* was present medially and ventrally along the ventricle (Figure 2G-H). Kctd12.1 and Kctd12.2 mark different populations of differentiated habenular neurons. At 72 hpf, neither showed any overlap with *dbx1b* expression (Figure 2I-L). Similar results were observed with another habenular differentiation marker, *pou4f1* (or *brn3a*) (data not shown). Together these results indicated that *dbx1b* expression is maintained in a proliferative population of habenular progenitor cells that reside along the ventricle. *Dbx1b* expression was absent in fully differentiated neurons, suggesting that *dbx1b* is downregulated in mature habenular neurons. Thus, we conclude that *dbx1b* can serve as an early marker to identify habenular progenitors.

**Figure 3:**
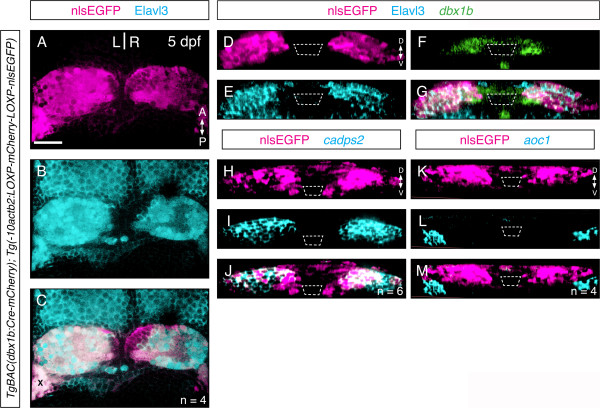
**Lineage labeling shows *dbx1b*-positive cells give rise to only dorsal habenular neurons. (A-C)** A *dbx1bBAC:cre* transgene lineage-labeled (magenta) nearly all Elav3l-positive neurons in the habenulae (cyan). See text for details. The bright area marked with X was due to trapped debris, not actually signal. **(D-G)** In the same lineage-labeling experiment, an Elav3l-negative domain corresponding the habenular progenitor domain, which is labeled by *dbx1b* expression (green), was clearly discernible as shown by coronal sections. **(H-J)** Lineage-labeled cells were restricted within the *cadps2*-positive dorsal habenular domain except right along the ventricle. **(K-M)** Lineage-labeled cells were excluded from the *aoc1*-positive ventral habenulae. Scale bars are 50 μm.

To substantiate our conclusion that *dbx1b* is expressed in habenular progenitors, we performed a lineage-tracing experiment using the Cre-lox recombination system. By crossing the *TgBAC[dbx1b:Cre-mCherry]*[21] transgenic line with a reporter line, *Tg[−10actb2:LOXP-mCherry-LOXP-nlsEGFP]*[22], almost all Elavl3-expressing habenular neurons were co-labeled with GFP (Figure 3A-C). Indeed, the only domain expressing GFP but not Elavl3 was found along the ventricle, in an area that coincides with *dbx1b* transcription (Figure 3D-G). To determine if *dbx1b*-positive cells give rise to both dorsal and ventral habenular neurons, we compared the lineage-labeled domain with *cadps2*, a dorsal habenular marker [23]. Almost all lineage-labeled cells were *cadps2*-positive (Figure 3H-J). The only *dbx1b*-derived cells (GFP-positive) outside the *cadps2* domain were medially located and along the ventricle; none were found laterally in the region of the ventral habenulae. Moreover, we also compared the lineage-labeled domain with the expression domain of *aoc1*, a known ventral habenular marker [24], and found that these two domains abutted but clearly did not overlap (Figure 3K-M). In conclusion, these results strongly suggested that most if not all post-mitotic dorsal habenular neurons are derived from progenitor cells that express *dbx1b* prior to their differentiation, confirming that *dbx1b* is a marker of early dorsal habenular progenitors.

### FGF signaling is required for proper development of the dorsal habenulae

**Figure 4:**
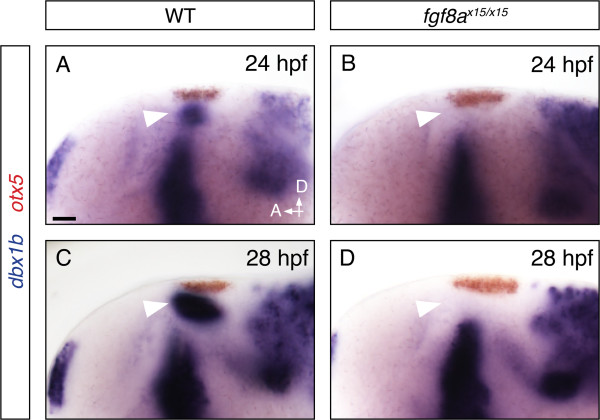
***Fgf8a *mutants fail to express *dbx1b *in the epithalamus. (A-D)***In situ* hybridization for *dbx1b* (blue) in wildtype and *fgf8a* mutant embryos. *otx5* marks the pineal complex. Scale bars are 10 μm.

FGF signaling has been shown to play critical roles during the development of the zebrafish dorsal diencephalon, particularly in pineal complex specification and parapineal migration [25,26]. It has been suggested that the development of both the left and right habenulae requires FGF signaling, as shown by the reduced expression of habenular differentiation markers (Kctd12.1, *pou4f1*) in FGF mutants [26]. However, how the loss of FGF signaling impacts habenular development remains unclear. We found that in *fgf8a* mutants, in which brain patterning appeared to be normal (Additional file 2), the expression of *dbx1b* was completely lost at 24 hpf (Figure 4A-D) and this loss of expression persisted at later stages. The latter observation suggested that the loss of FGF-dependent *dbx1b* is not a result of developmental delay. Therefore, FGF signaling is absolutely required for the initiation of *dbx1b* expression.

**Figure 5:**
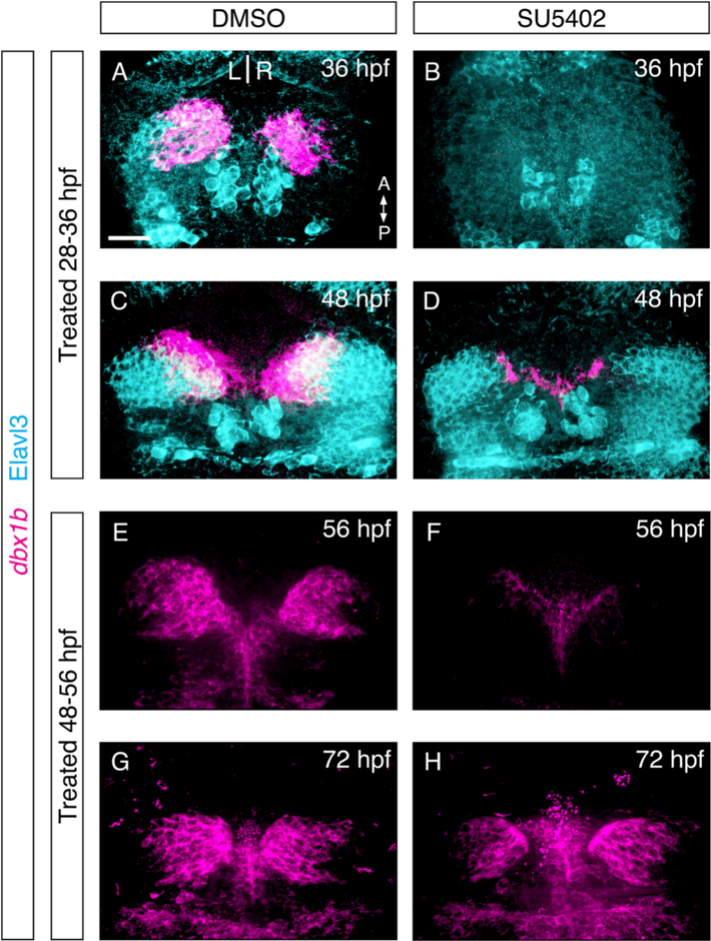
**Sustained fibroblast growth factor (FGF) signaling is required for *dbx1b *expression. (A-D)** Eight-hour treatment of embryos with the FGF receptor antagonist SU5402 abolished *dbx1b* expression, however expression began to return 12 hours after treatment. **(E-H)** Similar results were seen when FGF receptor blockade was initiated after *dbx1b* expression began. Generation of Elav3l-positive habenular cells resumed following drug washout at both early and late time points **(D, H)**. Scale bars are 50 μm.

To explore the regulatory relationship between FGF signaling and *dbx1b* in more detail, we investigated if FGF signaling is continuously required to maintain *dbx1b*-positive habenular progenitors. We took advantage of the FGF receptor antagonist, SU5402, to block FGF signaling in a temporally controlled manner. Since *dbx1b* expression was first detectable at 24 hpf, the embryos were treated with SU5402 for 8 hours, from either 28 to 36 hpf or 48 to 56 hpf. The expression of *dbx1b* was analyzed at the end of the treatments as well as after 12 hours of recovery post-treatment. As shown in Figure 5, both treatment regimes abrogated *dbx1b* expression by the end of the treatment window, yet *dbx1b* expression began to return after 12 hours of recovery (Figure 5B,D and 4F,H). This result suggests that FGF signaling is required for not only the initiation, but also the maintenance of *dbx1b* expression. Moreover, at least some neuronal progenitors in the dorsal diencephalon remain FGF-responsive and capable of reactivating *dbx1b* expression upon exposure to FGF signal, even when they were previously deprived of FGF signaling.

## Conclusions

This report describes the expression of *dbx1b*, which we believe is the first reported marker of the neuronal progenitors that give rise to the dorsal habenulae. In addition, we found that FGF signaling controls the expression of *dbx1b* in the dorsal diencephalon. Together with other existing genetic tools, including various *dbx1b* BAC transgenic lines, our discovery of *dbx1b* as a habenular progenitor marker will not only allow for more detailed and nuanced investigation of dorsal habenular development, but also provide an exciting way forward to study proliferation, specification and circuit formation of the diverse neuronal populations in the habenular nuclei, and how these processes influence developmental and adult habenulae-mediated behaviors.

## Methods

### Zebrafish maintenance and strains

Zebrafish were raised at 28.5°C on a 14/10 hour light/dark cycle and staged according to hours post-fertilization. The following fish lines were used: the wildtype strain AB*[27], *TgBAC[dbx1b:Cre-mCherry]*^*nns13a*^[21] and *Tg[−10actb2:LOXP-mCherry-LOXP-nlsEGFP]*^*pd31*^[22]. All experiments were approved by the Vanderbilt University’s Institutional Animal Care and Use Committee (IACUC) and Office of Animal Welfare, and performed according to national regulatory standards.

### Whole-mount *in situ* hybridization

Whole-mount RNA *in situ* hybridization was performed as described previously [23], with one change: 5% dextran sulfate was added to the hybridization buffer to enhance hybridization specificity [28]. Hybridized probes were detected using alkaline phosphatase conjugated antibodies (Roche, Indianapolis, IN, USA) and visualized by 4-nitro blue tetrazolium (NBT; Roche) and 5-bromo-4-chloro-3-indolyl-phosphate (BCIP; Roche) staining for single colorometric labeling, or by NBT/BCIP followed by iodonitrotetrazolium (INT) and BCIP staining for double colorometric labeling. *dbx1a* probe [17] was produced from pCRII-*dbx1a* plasmid linearized by EcoRV and transcribed by SP6 RNA polymerase. *dbx1b* probe [17] using pCRII-*dbx1b,* BamHI and T7 RNA polymerase, and *otx5*[29] using pBS-*otx5,* Not1 and T7 RNA polymerase.

### Whole-mount fluorescent *in situ* hybridization and immunohistochemistry

Whole-mount fluorescent *in situ* hybridization and immunohistochemical co-labeling was performed as described previously [13], with the following additional reagents: in addition to Fast Red substrate (F4648; Sigma-Aldrich, St Louis, MO, USA), some experiments used 3 × 5 minute washes in Fast Blue Buffer [28] and were developed in Fast Blue Substrate (0.25 mg/mL Fast Blue Substrate and 0.25 mg/mL nAMP in Fast Blue Buffer) diluted in Fast Blue Buffer. In addition to the anti-digoxigenin (DIG) antibody, the primary antibodies used were rabbit anti-pHH3 (1:500, EMD Millipore, Billerica, MA, USA), mouse anti-HuC (1:400, Life Technologies, Carlsbad, CA, USA), rabbit anti-GFP (1:500, Torrey Pines Biolab, Secaucus, NJ, USA), rabbit anti-Kctd12.1 and rabbit anti-Kctd12.2 (1:300, see [30]). Primary antibody was detected using goat-anti-rabbit or goat-anti-mouse antibodies conjugated to Alexa 488, Alexa 568 or Alexa 633 fluorophores (1:300, Molecular Probes, Eugene, OR, USA).

Double fluorescent *in situ* hybridization was performed with the following modifications to the above colorometric *in situ* protocol: after hybridization of DIG and fluorescein-labeled probes, anti-DIG antibody was applied (1:5,000, Roche) overnight at 4°C. The following day, embryos were washed 4 × 20 minutes in PBS with Triton (PBSTr) and 3 × 5 minutes in Fast Blue Buffer and developed in Fast Blue Substrate diluted in Fast Blue Buffer. After color development, embryos were washed 2 × 10 minutes in PBSTr. The alkaline phosphatase was acid inactivated by a 10-minute wash in 0.1 M glycine HCl, pH 2.0. After 2 × 10 minute PBSTr washes, embryos were incubated in anti-fluorescein antibody (1:1,000, Roche) overnight at 4°C. The following day, color was developed in Fast Red substrate as in Doll *et al*. [13]. *cxcr4b*[31] probe was generated with EcoRV and SP6 RNA polymerase.

### Inhibitor treatments

For whole-mount *in situ* hybridizations and antibody labeling, embryos were incubated in their chorions in 12 μM (for complete receptor inhibition; [27]) of SU5402 (Tocris Bioscience, Bristol, UK) dissolved in 0.3% dimethyl sulfoxide (DMSO) in egg water supplemented with 0.003% N-phenylthiourea (PTU; Sigma-Aldrich) to prevent melanin formation. Control embryos were treated with 0.3% DMSO in parallel with their SU5402-treated siblings. Embryos were either fixed immediately following treatment or SU5402/DMSO was washed off with 5 × 5 minute egg water before being returned to egg water with PTU to develop to the desired stage for fixation.

### Imaging

All samples were cleared in a glycerol series (50%, 100%). Colorometric *in situ* images were captured on a Leica DM6000 B compound microscope (Leica Microsystems, Buffalo Grove, IL, USA) under a 20× air objective in bright field conditions. Fluorescent images were captured on a PerkinElmer spinning disk confocal microscope (PerkinElmer, Waltham, MA, USA) or a Zeiss LSM 510 Meta confocal microscope (Carl Zeiss Microscopy, Oberkochen, Germany) with a 40× oil-immersion objective and analyzed with Volocity software (Improvision/PerkinElmer, Waltham, MA, USA).

## Abbreviations

actb2: actin, beta 2; ADHD: attention deficit hyperactivity disorder; aoc1: amine oxidase, copper containing 1; BAC: bacterial artificial chromosome; BCIP: 5-bromo-4-chloro-3-indolyl-phosphate; cadps2: Ca^2+^-dependent secretion activator 2; cxcr4b: chemokine (C-X-C motif), receptor 4b; dbx1b: developing brain homeobox 1b; DIG: digoxigenin; dpf: days post-fertilization; DMSO: dimethyl sulfoxide; Elavl3: ELAV like neuron-specific RNA binding protein 3; eomesa: eomesodermin homolog a; FGF: fibroblast growth factor; GFP: green fluorescent protein; hpf: hours post-fertilization; HuC: Hu-antigen C; IACUC: Institutional Animal Care and Use Committee; Kctd12: potassium channel tetramerization domain containing 12; NBT: 4-nitro blue tetrazolium; INT: iodonitrotetrazolium; PBS: phosphate-buffered saline; otx5: orthodenticle homolog 5; pou4f1: POU domain, class 4, transcription factor 1 (also called Brain-specific homeobox/POU domain protein 3a, *brn3a*); PTU: N-phenylthiourea; shh: sonic hedgehog; TFs: transcription factors; Tg: transgenic; wnt1: wingless-type MMTV integration site family, member 1; ZLI: zona limitans intrathalamica

## Competing interests

The authors declare that they have no competing interests.

## Authors’ contributions

BD conceived and designed the study, undertook data collection and analysis and wrote the manuscript. BE undertook data collection. JG conceived and designed the study, arranged financial support and wrote the manuscript. SW conceived and designed the study, undertook data collection and analysis, and wrote the manuscript. All authors read and approved the final manuscript.

## Supplementary Material

Additional file 1 ***Dbx1b *** **is expressed in the dorsal diencephalon.** Lateral and dorsal views of a 28 hpf wildtype embryo. (A and B) *In situ* hybridization for *dbx1a* (blue) revealed several expression domains, including the olfactory bulb (Ob), prethalamus (pT), thalamus (Th), and midbrain (Mb) throughout the brain, but no expression in the dorsal diencephalon (arrow heads). (C and D) *dbx1b* transcript (blue) was expressed in a similar pattern but with greatly reduced expression in thalamus and robust expression in the dorsal diencephalon and olfactory bulb. *otx5* (red) marks the pineal complex (P), a component of the dorsal diencephalon. Scale bars are 10 μm. Click here for file

Additional file 2 ***Fgf8a *** **mutants show normal overall brain patterning.** (A-B) In *fgf8a* mutants there were no major anterior-posterior patterning defects observed. *eomes*, *shh* and *wnt1* mark the telencephalon (Tel), zona limitans intrathalamica (ZLI) and midbrain (Mb) respectively. *otx5* marks the pineal complex. (C-D) Dorsal-ventral patterning was also unaffected in *fgf8a* mutants. *wnt3a* (blue) marks the ZLI and midbrain and *shh* (red) marks the ZLI. Scale bars are 10 μm. Click here for file
