# Supplementary figures and images for: Dbx1b defines the dorsal habenular progenitor domain in the zebrafish epithalamus
Source: Neural Dev. 2014 Sep 12;9:20. doi: 10.1186/1749-8104-9-20 (PMC4164515; doi:10.1186/1749-8104-9-20)

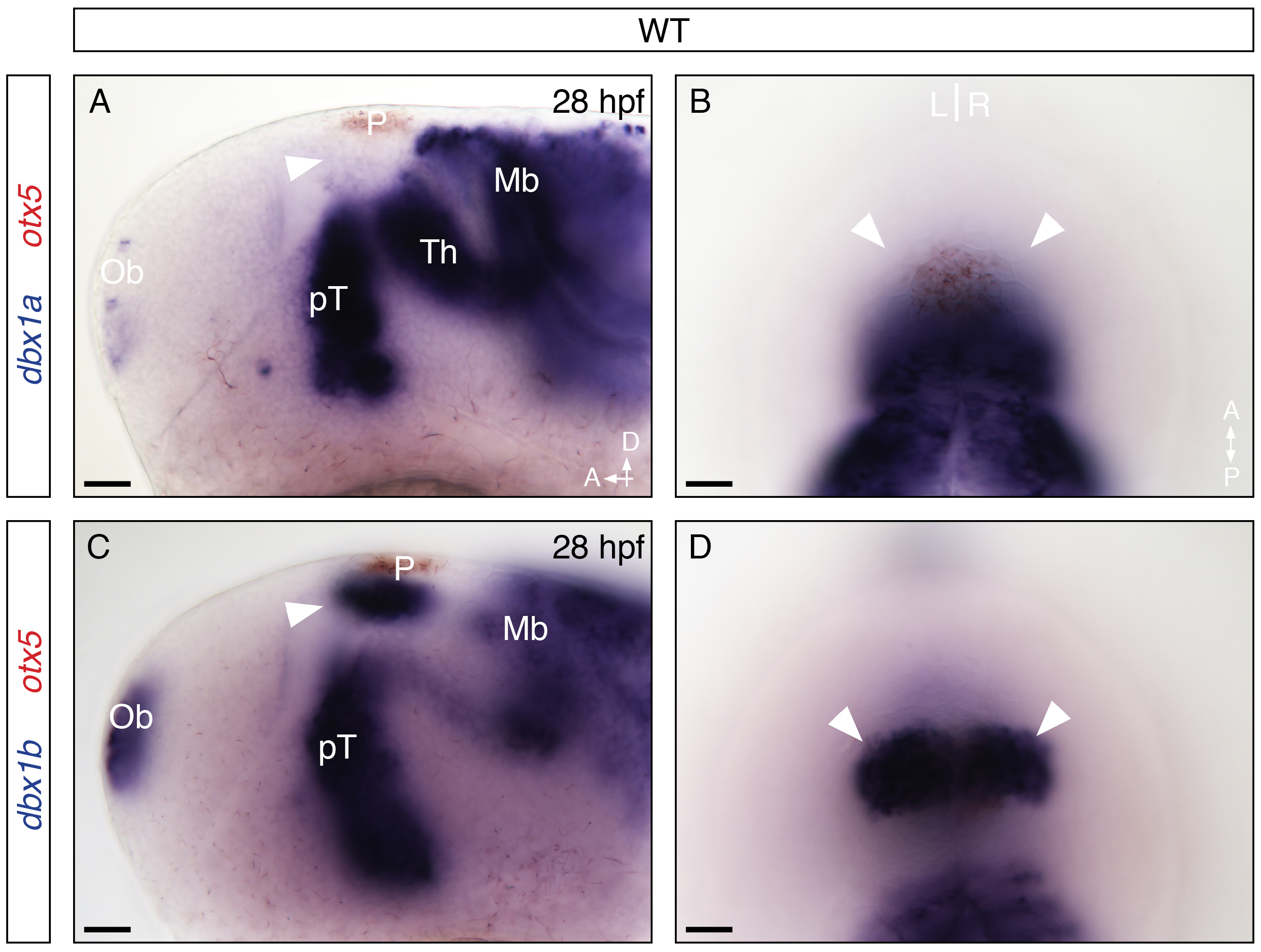

Supplement: Additional file 1 — Dbx1b is expressed in the dorsal diencephalon. Lateral and dorsal views of a 28 hpf wildtype embryo. (A and B) In situ hybridization for dbx1a (blue) revealed several expression domains, including the olfactory bulb (Ob), prethalamus (pT), thalamus (Th), and midbrain (Mb) throughout the brain, but no expression in the dorsal diencephalon (arrow heads). (C and D) dbx1b transcript (blue) was expressed in a similar pattern but with greatly reduced expression in thalamus and robust expression in the dorsal diencephalon and olfactory bulb. otx5 (red) marks the pineal complex (P), a component of the dorsal diencephalon. Scale bars are 10 μm. [file 1749-8104-9-20-S1.jpeg]

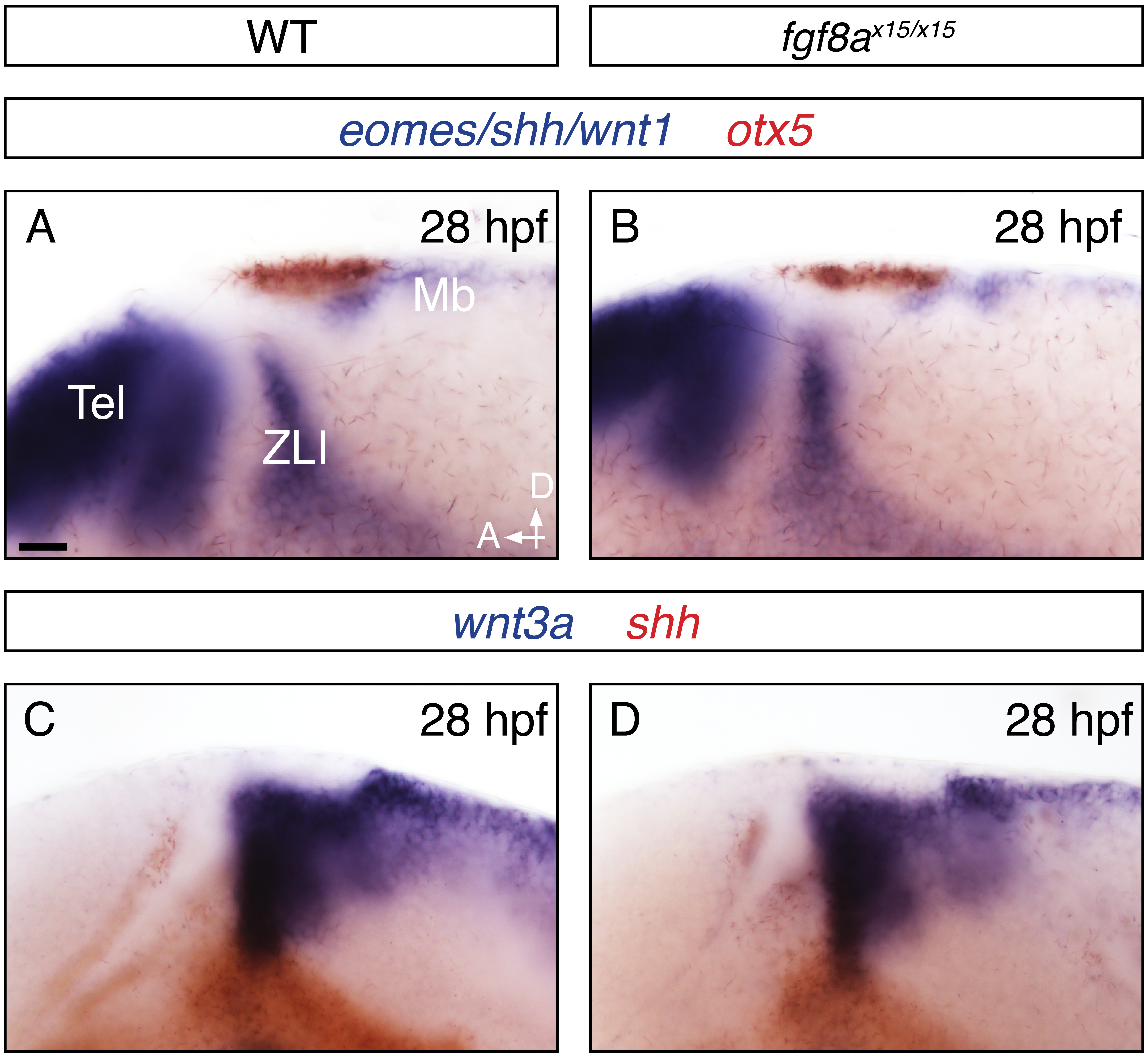

Supplement: Additional file 2 — Fgf8a mutants show normal overall brain patterning. (A-B) In fgf8a mutants there were no major anterior-posterior patterning defects observed. eomes, shh and wnt1 mark the telencephalon (Tel), zona limitans intrathalamica (ZLI) and midbrain (Mb) respectively. otx5 marks the pineal complex. (C-D) Dorsal-ventral patterning was also unaffected in fgf8a mutants. wnt3a (blue) marks the ZLI and midbrain and shh (red) marks the ZLI. Scale bars are 10 μm. [file 1749-8104-9-20-S2.jpeg]
